# Lactate-avid regulatory T cells: metabolic plasticity controls immunosuppression in tumour microenvironment

**DOI:** 10.1038/s41392-021-00598-0

**Published:** 2021-04-30

**Authors:** Gabriele Multhoff, Peter Vaupel

**Affiliations:** 1 Radiation Immuno-Oncology Project Group, TranslaTUM – Central Institute for Translational Cancer Research, Klinikum rechts der Isar, TU München, Munich, Germany; 2 Department of Radiation Oncology, Klinikum rechts der Isar, TU München, Munich, Germany; 3 Department of Radiooncology and Radiotherapy, Tumour Pathophysiology Group, University Medical Centre Mainz, Mainz, Germany

**Keywords:** Preclinical research, Tumour immunology

**Fig. 1:**
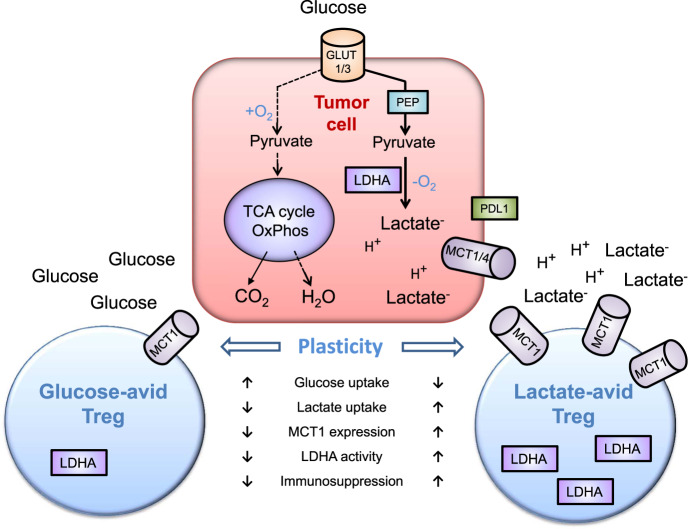
Effects of glycolysis-derived extracellular lactic acid on the plasticity of regulatory T (Treg) cells. To maintain energy homoeostasis, tumour cells have an enhanced glycolytic flux. Glucose import is mediated by an overexpression of GLUT1/3 transporters under normoxic (Warburg effect) and hypoxic (anaerobic glycolysis) conditions. Especially under hypoxia glucose is metabolized via phosphoenolpyruvate (PEP) into pyruvate by glycolysis and does not enter the tricarboxylic acid (TCA, Krebs) cycle and undergoes oxidative phosphorylation (OxPhos). Pyruvate is converted into lactate anion (Lactate^-^) and gets imported/exported via MCT1/4 symporters. High extracellular lactate^-^ levels contribute to an acidification (pH <6.9) of the tumour microenvironment (TME), stimulate the expression of the immune checkpoint inhibitor ligand PDL1 on tumour cells, and serve as a fuel for lactate-avid Treg cells. Lactate-avid Treg cells express higher numbers (associated with a higher activity) of the lactate importer MCT1, show an enhanced lactate dehydrogenase A (LDHA) activity, and immunosuppressive capacity compared to glucose-avid Treg cells. In this simplified scheme only transporters and enzymes relevant for the highlight report are illustrated. (GLUT1/3 glucose-transporter 1/3, LDHA lactate dehydrogenase A, MCT1/4 lactate importer/exporter, OxPhos oxidative phosphorylation, PEP, phosphoenolpyruvate, PDL1 immune checkpoint inhibitor ligand of PD-1, TCA tricarboxylic acid, TME tumour microenvironment.)

The excellent article by Watson et al.^1^ points the way ahead to the important role of lactic acid/lactate^-^ anion and glucose on the immunosuppressive activity of lactate- and glucose-affine metabolically distinct subsets of regulatory T (Treg) cells within the tumour microenvironment (TME) (Fig. 1).

The glycolytic (Warburg) phenotype observed in 70–80% of human cancers which develops early in carcinogenesis without mitochondrial dysfunction, is a major component of a metabolic reprogramming (aerobic glycolysis) that enables a survival benefit in many solid tumours. Among many other processes, the Warburg effect accelerates glycolytic fluxes, rapidly produces adequate amounts of ATP, inhibits entry of pyruvate into mitochondria and leads to a tremendous accumulation of the oncometabolite lactate inside the tumour and in the tumour microenvironment (TME).^2^ High extracellular lactate (lactate^-^ anion) levels allow the cancer cells to escape from protective anti-tumour immune responses and support immunosuppressive mechanisms. Due to an immature, chaotic microvasculature most solid tumours are hypoxic and hypoxia-inducible factor (HIF)-driven downstream transcription factors, including adenosine (up to 100 µM), vascular endothelial growth factor (VEGF), VEGF-receptor (VEGFR) and lactate (up to 40 mM) are further supporting these immunosuppressive activities.^2^ Extracellular acidosis (pH <6.9) induced by increased lactic acid levels^1^ as a consequence of an upregulated glycolysis impairs monocyte differentiation and antigen presentation via epigenetic reprogramming, inhibits the activity of CD8^+^ cytotoxic T cells and NK cells and recruits immunosuppressive cells including M2 macrophages, N2 tumour-associated neutrophils, myeloid-derived suppressor cells (MDSCs) and Treg cells to the acidic TME. Indoleamine-2,3-dioxygenase in Treg cells converts tryptophan to kynurenine and thereby activates stress responses.^3^

In healthy individuals, Treg cells which were previously characterized as a homogenous cell population expressing CD4^+^, CD25^+^ and Forkhead box P3 (FoxP3^+^) are intimately involved in the regulation of the peripheral self-tolerance and thereby avoid autoimmunity. In tumour patients, however, Treg cells foster immune tolerance by impairing protective anti-cancer immune responses.

The article of Watson et al.^1^ elegantly demonstrates the existence of at least two different subsets of Treg cells that are endowed with a metabolic flexibility to preferentially use either glucose or lactate as carbon sources. Depending on the availability of the carbon source, glucose-affine Treg cells appear to differ significantly from lactate-affine Treg cells in many functions including their pro-tumour and immunosuppressive capacities. Despite the classical Treg signature (FoxP3^+^, CD25^+^, Nrp1^+^) and the expression of the lactate-proton importer MCT1, Treg cells with a high avidity to glucose are less immunosuppressive and pro-tumourigenic than those with a high lactate avidity. This was convincingly illustrated by Watson et al.^1^ by extensive in vitro studies of Treg cells derived from different sources and preclinical tumour- as well as colitis-induced autoimmunity models. It has further been shown that Treg cells are metabolically ‘out-of-syn’ since they need to be active when other immune effector cells are resting. Thereby, Treg cells acquired mechanisms to metabolize lactate to fuel their metabolic demand in times of starvation and/or nutrient deficiency.

In support of the pro-tumourigenic activity of lactate (lactate^-^ anion) on tumour cells, the authors demonstrate that lactate-avid Treg cells promote the production of the immunosuppressive cytokine IL-10, the stem cell factor CD44 supporting epithelial-mesenchymal transition and EGFR signalling and the pro-angiogenic co-receptor Nrp1. The presence of MCT1-deficient, glucose-avid Treg cells not only decreases tumour growth, but also maintains the immunocompetence of terminally differentiated cytotoxic (CD8^+^) and conventional (CD4^+^) T cells with an elevated PD-1 and Tim3 expression. Therefore, lowering the lactate availability for Treg cells in the TME might synergistically support immune checkpoint inhibitor therapies as well as immune effector cell based therapeutic concepts (e.g. ex vivo Hsp70-activated NK cells after radiochemotherapy).^4^

The authors further highlight the antagonistic effects of a high glucose availability on lactate-affine Treg cells. Treg cells per se are not dependent on lactate, but are endowed to metabolize lactate in case of emergency or in hypoxic tumour microenvironments. Due to the described Treg cell plasticity the authors show that high concentrations of glucose (25 mM) which are not physiologically relevant (since only seen in severe hyperglycemia) can reverse a lactate-affine Treg cell with a high immunosuppressive, tumourigenic capacity into a glucose-affine Treg cell with a lower immunosuppressive and pro-tumour potential. With respect to the findings based on a greatly increased availability of glucose it needs to be mentioned that glucose supply in cancer tissues in vivo is restricted due to perfusion and diffusion limitations. As a consequence, glucose levels in solid tumours usually are <2.5 mM with average values around 1 mM.^2^

The article is highlighting novel functions of Treg cell subsets which are depending on different metabolic resources. This is highly relevant in the TME especially under hypoxic conditions with very high lactate levels. Regarding this aspect it would be of great interest to study the impact of hypoxia (pO_2_ < 7 mmHg or cO_2_ < 1%) on the glucose- and lactate-affine Treg cell subsets.

Apart from very high glucose concentrations in the TME or a MCT1-inhibition as suggested by the authors,^1^ an impairment of the lactate metabolism or transport^3^ might also exert beneficial effects on the immunosuppressive barrier of Treg cells and other immunosuppressive cells such as tumour-associated M2 macrophages that have also been shown to utilize lactate as a fuel. A recent report indicates that diclofenac, but not other non-steroidal anti-inflammatory drugs can impair the lactate import/export of tumour cells via MCT1 and MCT4 transporters.^5^ Therefore, it would be interesting to assess putative beneficial effects of diclofenac on lactate-affine Treg cells.
